# Butyl­bis­(dimethyl­glyoximato-κ^2^ *N*,*N*′)(pyridine-κ*N*)cobalt(III)^1^

**DOI:** 10.1107/S1600536812000967

**Published:** 2012-01-14

**Authors:** Sarvendra Kumar, Suresh Thapa

**Affiliations:** a DQIAQF/INQUIMAE, Universidad de Buenos Aires, Ciudad Universitaria, Pab. II, p. 3, EHA1428 Buenos Aires, Argentina; b Faculty of Science and Technology, Purbanchal University, Biratnagar, Nepal

## Abstract

In the title compound, [Co(C_4_H_9_)(C_4_H_7_N_2_O_2_)_2_(C_5_H_5_N)], which was prepared as a model complex of vitamin B_12_, the Co^III^ atom is coordinated by a butyl group, a pyridine and two *N*,*N*′-bidentate dimethyl­glyoximate ligands in a distorted octa­hedral geometry. The bis-chelating dimethyl­glyoximate ligands, which occupy equatorial sites, are linked by strong intra­molecular O—H⋯O hydrogen bonds.

## Related literature

For general background to organocobaloximes, see: Schrauzer & Kohnle (1964 ▶); Schrauzer (1968 ▶, 1976 ▶). For applications of cobaloximes, see: Rockenbaur *et al.* (1982 ▶); Giese (1986 ▶). For structure–property relationships of cobaloximes, see: Gupta *et al.* (2004 ▶). For related structures, see: Mandal & Gupta (2005 ▶, 2007 ▶); Kumar & Gupta (2011 ▶).
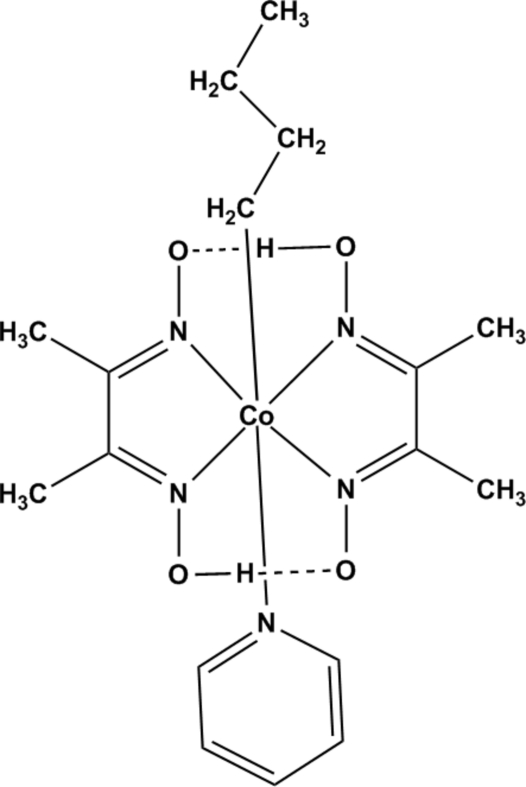


## Experimental

### 

#### Crystal data

[Co(C_4_H_9_)(C_4_H_7_N_2_O_2_)_2_(C_5_H_5_N)]
*M* *_r_* = 425.37
Monoclinic, 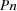

*a* = 8.365 (2) Å
*b* = 10.408 (2) Å
*c* = 11.487 (3) Å
β = 91.768 (4)°
*V* = 999.7 (4) Å^3^
*Z* = 2
Mo *K*α radiation
μ = 0.89 mm^−1^
*T* = 100 K
0.22 × 0.18 × 0.16 mm

#### Data collection

Bruker SMART CCD area-detector diffractometer
Absorption correction: multi-scan (*SADABS*; Bruker, 2001 ▶) *T*_min_ = 0.828, *T*_max_ = 0.871
5174 measured reflections
3400 independent reflections
3144 reflections with *I* > 2σ(*I*)
*R*_int_ = 0.031

#### Refinement

*R*[*F* ^2^ > 2σ(*F* ^2^)] = 0.042
*wR*(*F* ^2^) = 0.100
*S* = 1.05
3400 reflections
256 parameters
4 restraints
H atoms treated by a mixture of independent and constrained refinement
Δρ_max_ = 0.90 e Å^−3^
Δρ_min_ = −0.25 e Å^−3^
Absolute structure: Flack (1983 ▶), 1551 Friedel pairs
Flack parameter: 0.038 (16)

### 

Data collection: *SMART* (Bruker, 2001 ▶); cell refinement: *SAINT* (Bruker, 2001 ▶); data reduction: *SAINT*; program(s) used to solve structure: *SHELXS97* (Sheldrick, 2008 ▶); program(s) used to refine structure: *SHELXL97* (Sheldrick, 2008 ▶); molecular graphics: *SHELXTL* (Sheldrick, 2008 ▶); software used to prepare material for publication: *DIAMOND* (Brandenburg, 1999 ▶).

## Supplementary Material

Crystal structure: contains datablock(s) I, global. DOI: 10.1107/S1600536812000967/is5041sup1.cif

Structure factors: contains datablock(s) I. DOI: 10.1107/S1600536812000967/is5041Isup2.hkl

Additional supplementary materials:  crystallographic information; 3D view; checkCIF report

## Figures and Tables

**Table 1:** Selected bond lengths (Å)

N1—Co1	1.887 (4)
N2—Co1	1.884 (4)
N3—Co1	1.873 (4)
N4—Co1	1.881 (4)
N5—Co1	2.061 (4)

**Table 2:** Hydrogen-bond geometry (Å, °)

*D*—H⋯*A*	*D*—H	H⋯*A*	*D*⋯*A*	*D*—H⋯*A*
O1—H1⋯O4	0.86 (2)	1.61 (2)	2.465 (3)	174 (6)
O3—H2⋯O2	0.87 (2)	1.60 (2)	2.465 (3)	171 (6)

**Fig. 1.**
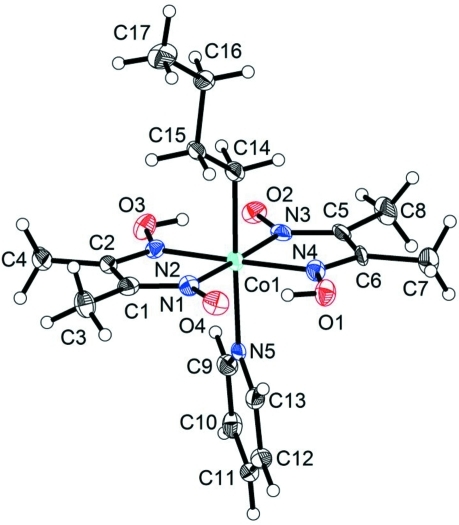
ORTEP diagram of the title compound with 50% probability displacement ellipsoids for non-hydrogen atoms.

**Fig. 2.**
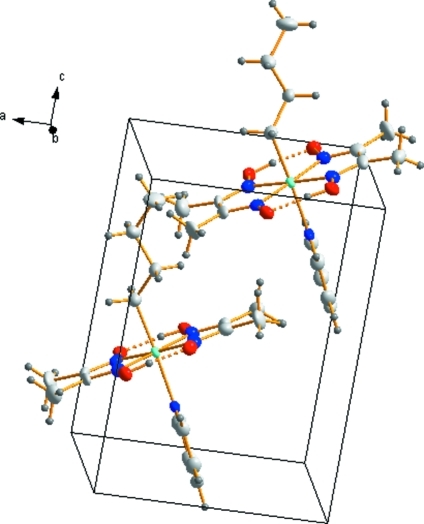
Packing diagram of the title compound in the unit cell. Dashed lines indicate the O—H···O hydrogen bonds.

## supplementary crystallographic information

### Comment

Organocobaloximes have extensively been used as structural and functional mimic for vitamin B_12_ ever since these were first introduced by Schrauzer four decades ago as model of vitamin B_12_ (Schrauzer & Kohnle, 1964; Schrauzer, 1968, 1976). These represent a unique class of compounds in organometallic and bioinorganic chemistry. These have rich coordination chemistry, application in organic synthesis and catalysis, and their stability and redox properties are of particular interest (Rockenbaur *et al.*, 1982; Giese, 1986). The general formula of cobaloximes is RCo(L)B, where R is an organic group, bonded to cobalt, B is an axial base *trans* to the organic group, and L is a monoanionic dioxime ligand. Dimethylglyoximate (dmg) is a familiar ligand with excellent coordination capability to generate mono-, bi- or trinuclear complexes. Cobaloximes are best characterized by NMR and X-ray studies. Most of the recent studies on cobaloximes have been focused on their structure–property relationships (Gupta *et al.*, 2004). We synthesized the title compound and determined its structure.

In the title compound, the cobalt atom is in a distorted octahedral geometry (Table 1) by four N atoms of the *N*,*N*-bidentate dimethylglyoximate ligands in the equatorial plane, and by a butyl group and a nitrogen atom of pyridine in mutually trans positions [N5—Co1—C14 = 177.15 (16)°; Fig. 1]. The Co—N(dmg) bonds range in length from 1.872 (3) to 1.887 (3) Å. The plane of the four nitrogen atoms is particular planar. The O—H···O bridge (Table 2) in the structure is very common in cobaloxime derivatives (Mandal & Gupta, 2005, 2007; Kumar & Gupta, 2011). In packing diagram (Fig. 2), one unit cell contains two molecules.

### Experimental

A solution of ClCo(dmgH)_2_py (1 mmol) in 10 ml of methanol was purged thoroughly with N_2_ for 20 min and was cooled to 0 °C with stirring. The solution turned deep blue after the addition of a few drops of aqueous NaOH followed by sodium borohydride (1.5 mmol in 0.5 ml of water). The color of the solution turned orange-red on the addition of bromobutane (1.5 mmol). The reaction was stirred 1 h at 0 °C then poured into 20 ml chilled water. The resulting orange-red precipitate was filtered, washed with water, and dried. The crude product was purified on the silica gel column using dichloromethane. The obtained orange colored compound was recrystallized from dichloromethane and methanol. After three days, orange colored crystals obtained which were suitable for single-crystal data collection.

### Refinement

Atoms H1 and H2 were located in a difference Fourier map and were refined with the O—H distance restraints of 0.84 (2) Å. Other hydrogen atoms were placed in calculated positions and included in the refinement in a riding-model approximation, with C—H = 0.95–0.99 Å, and with *U*_iso_(H) = 1.2*U*_eq_(C) or 1.5*U*_eq_(methyl C).

### Crystal data

**Table:**

[Co(C_4_H_9_)(C_4_H_7_N_2_O_2_)_2_(C_5_H_5_N)]	*F*(000) = 448
*M**_r_* = 425.37	*D*_x_ = 1.413 Mg m^−^^3^
Monoclinic, *P**n*	Mo *K*α radiation, λ = 0.71073 Å
Hall symbol: P -2yac	Cell parameters from 1911 reflections
*a* = 8.365 (2) Å	θ = 2.6–28.2°
*b* = 10.408 (2) Å	µ = 0.89 mm^−^^1^
*c* = 11.487 (3) Å	*T* = 100 K
β = 91.768 (4)°	Prism, orange
*V* = 999.7 (4) Å^3^	0.22 × 0.18 × 0.16 mm
*Z* = 2	

### Data collection

**Table:**

Bruker SMART CCD area-detector diffractometer	3400 independent reflections
Radiation source: fine-focus sealed tube	3144 reflections with *I* > 2σ(*I*)
graphite	*R*_int_ = 0.031
φ and w scans	θ_max_ = 25.5°, θ_min_ = 2.6°
Absorption correction: multi-scan (*SADABS*; Bruker, 2001)	*h* = −10→10
*T*_min_ = 0.828, *T*_max_ = 0.871	*k* = −12→8
5174 measured reflections	*l* = −13→13

### Refinement

**Table:**

Refinement on *F*^2^	Secondary atom site location: difference Fourier map
Least-squares matrix: full	Hydrogen site location: inferred from neighbouring sites
*R*[*F*^2^ > 2σ(*F*^2^)] = 0.042	H atoms treated by a mixture of independent and constrained refinement
*wR*(*F*^2^) = 0.100	*w* = 1/[σ^2^(*F*_o_^2^) + (0.048*P*)^2^] where *P* = (*F*_o_^2^ + 2*F*_c_^2^)/3
*S* = 1.05	(Δ/σ)_max_ < 0.001
3400 reflections	Δρ_max_ = 0.90 e Å^−^^3^
256 parameters	Δρ_min_ = −0.25 e Å^−^^3^
4 restraints	Absolute structure: Flack (1983), 1551 Friedel pairs
Primary atom site location: structure-invariant direct methods	Flack parameter: 0.038 (16)

### Special details

**Table:**

Geometry. All s.u.'s (except the s.u. in the dihedral angle between two l.s. planes) are estimated using the full covariance matrix. The cell s.u.'s are taken into account individually in the estimation of s.u.'s in distances, angles and torsion angles; correlations between s.u.'s in cell parameters are only used when they are defined by crystal symmetry. An approximate (isotropic) treatment of cell s.u.'s is used for estimating s.u.'s involving l.s. planes.
Refinement. Refinement of *F*^2^ against ALL reflections. The weighted *R*-factor *wR* and goodness of fit *S* are based on *F*^2^, conventional *R*-factors *R* are based on *F*, with *F* set to zero for negative *F*^2^. The threshold expression of *F*^2^ > 2σ(*F*^2^) is used only for calculating *R*-factors(gt) *etc*. and is not relevant to the choice of reflections for refinement. *R*-factors based on *F*^2^ are statistically about twice as large as those based on *F*, and *R*- factors based on ALL data will be even larger.

### Fractional atomic coordinates and isotropic or equivalent isotropic displacement parameters (Å^2^)

**Table:**

	*x*	*y*	*z*	*U*_iso_*/*U*_eq_	
C1	0.5829 (5)	0.3423 (5)	0.8074 (3)	0.0196 (11)	
C2	0.5910 (5)	0.2031 (5)	0.8137 (4)	0.0211 (11)	
C3	0.7089 (6)	0.4250 (5)	0.7546 (4)	0.0340 (13)	
H3A	0.7145	0.4059	0.6713	0.051*	
H3B	0.8127	0.4073	0.7931	0.051*	
H3C	0.6815	0.5157	0.7650	0.051*	
C4	0.7261 (5)	0.1253 (5)	0.7673 (4)	0.0334 (12)	
H4A	0.7155	0.0357	0.7922	0.050*	
H4B	0.8282	0.1600	0.7974	0.050*	
H4C	0.7229	0.1294	0.6820	0.050*	
C5	0.0468 (5)	0.1913 (5)	1.0286 (4)	0.0224 (11)	
C6	0.0360 (5)	0.3328 (5)	1.0188 (3)	0.0218 (12)	
C7	−0.0980 (6)	0.4091 (5)	1.0639 (4)	0.0284 (12)	
H7A	−0.0928	0.4970	1.0336	0.043*	
H7B	−0.0903	0.4110	1.1492	0.043*	
H7C	−0.1998	0.3698	1.0388	0.043*	
C8	−0.0731 (6)	0.1105 (5)	1.0866 (4)	0.0323 (12)	
H8A	−0.0743	0.0245	1.0518	0.048*	
H8B	−0.1792	0.1496	1.0764	0.048*	
H8C	−0.0451	0.1039	1.1698	0.048*	
C9	0.1611 (4)	0.1295 (4)	0.7124 (3)	0.0202 (8)	
H9	0.1899	0.0561	0.7576	0.024*	
C10	0.0893 (4)	0.1114 (4)	0.6052 (3)	0.0235 (8)	
H10	0.0691	0.0272	0.5765	0.028*	
C11	0.0463 (5)	0.2182 (4)	0.5392 (3)	0.0234 (9)	
H11	−0.0039	0.2082	0.4644	0.028*	
C12	0.0774 (5)	0.3386 (4)	0.5834 (3)	0.0237 (8)	
H12	0.0491	0.4133	0.5399	0.028*	
C13	0.1511 (4)	0.3484 (4)	0.6931 (3)	0.0222 (8)	
H13	0.1725	0.4316	0.7238	0.027*	
C14	0.4381 (5)	0.2916 (4)	1.0654 (4)	0.0230 (10)	
H14A	0.5480	0.2602	1.0529	0.028*	
H14B	0.4463	0.3853	1.0789	0.028*	
C15	0.3839 (4)	0.2318 (4)	1.1776 (3)	0.0197 (8)	
H15A	0.3773	0.1374	1.1682	0.024*	
H15B	0.2757	0.2640	1.1949	0.024*	
C16	0.4999 (5)	0.2642 (4)	1.2799 (3)	0.0255 (9)	
H16A	0.6055	0.2256	1.2650	0.031*	
H16B	0.5139	0.3585	1.2840	0.031*	
C17	0.4431 (6)	0.2162 (4)	1.3960 (3)	0.0351 (10)	
H17A	0.5216	0.2394	1.4576	0.053*	
H17B	0.4312	0.1226	1.3933	0.053*	
H17C	0.3398	0.2556	1.4124	0.053*	
N1	0.4540 (4)	0.3883 (4)	0.8523 (3)	0.0197 (9)	
N2	0.4708 (4)	0.1519 (4)	0.8647 (3)	0.0198 (9)	
N3	0.1770 (4)	0.1474 (4)	0.9832 (3)	0.0186 (9)	
N4	0.1586 (4)	0.3816 (4)	0.9692 (3)	0.0174 (9)	
N5	0.1929 (5)	0.2471 (3)	0.7568 (3)	0.0181 (8)	
O1	0.1700 (4)	0.5107 (3)	0.9556 (2)	0.0255 (8)	
O2	0.2086 (4)	0.0191 (3)	0.9865 (2)	0.0259 (8)	
O3	0.4583 (4)	0.0245 (3)	0.8781 (3)	0.0266 (8)	
O4	0.4259 (4)	0.5151 (3)	0.8549 (2)	0.0242 (8)	
Co1	0.31315 (6)	0.26668 (4)	0.91509 (5)	0.01707 (14)	
H1	0.261 (4)	0.517 (5)	0.923 (5)	0.072 (19)*	
H2	0.374 (5)	0.016 (6)	0.921 (5)	0.09 (2)*	

### Atomic displacement parameters (Å^2^)

**Table:**

	*U*^11^	*U*^22^	*U*^33^	*U*^12^	*U*^13^	*U*^23^
C1	0.010 (2)	0.034 (3)	0.015 (2)	−0.006 (2)	−0.0007 (17)	0.000 (2)
C2	0.008 (2)	0.041 (3)	0.014 (2)	0.004 (2)	0.0023 (17)	−0.001 (2)
C3	0.022 (3)	0.051 (3)	0.029 (2)	−0.011 (2)	0.011 (2)	0.007 (2)
C4	0.024 (3)	0.045 (3)	0.031 (3)	0.005 (2)	0.006 (2)	−0.004 (2)
C5	0.022 (3)	0.030 (3)	0.016 (2)	−0.003 (2)	−0.0005 (18)	−0.003 (2)
C6	0.014 (2)	0.035 (3)	0.016 (2)	−0.001 (2)	0.0005 (17)	−0.005 (2)
C7	0.019 (2)	0.044 (3)	0.022 (2)	0.005 (2)	−0.0013 (19)	−0.005 (2)
C8	0.021 (2)	0.044 (3)	0.033 (3)	−0.011 (2)	0.0120 (19)	0.000 (2)
C9	0.019 (2)	0.019 (2)	0.0230 (19)	0.0007 (15)	0.0031 (15)	−0.0017 (15)
C10	0.021 (2)	0.022 (2)	0.028 (2)	−0.0009 (16)	0.0005 (16)	−0.0066 (16)
C11	0.016 (2)	0.035 (2)	0.0193 (19)	−0.0010 (18)	0.0008 (16)	−0.0064 (17)
C12	0.022 (2)	0.025 (2)	0.0245 (19)	0.0074 (17)	0.0038 (15)	0.0073 (17)
C13	0.019 (2)	0.022 (2)	0.026 (2)	−0.0011 (16)	0.0002 (16)	−0.0016 (16)
C14	0.016 (2)	0.034 (3)	0.018 (2)	0.000 (2)	−0.0008 (16)	0.0003 (19)
C15	0.0129 (19)	0.028 (2)	0.0178 (19)	−0.0011 (15)	−0.0008 (15)	0.0010 (15)
C16	0.021 (2)	0.038 (3)	0.017 (2)	−0.0047 (19)	0.0017 (17)	−0.0026 (17)
C17	0.044 (3)	0.044 (3)	0.017 (2)	−0.002 (2)	−0.0005 (18)	0.0068 (18)
N1	0.0166 (19)	0.029 (3)	0.0132 (17)	−0.0046 (16)	−0.0033 (14)	0.0018 (15)
N2	0.018 (2)	0.023 (2)	0.0180 (17)	0.0051 (16)	−0.0003 (14)	0.0023 (14)
N3	0.019 (2)	0.021 (2)	0.0160 (17)	0.0008 (15)	0.0001 (14)	0.0036 (14)
N4	0.0196 (19)	0.018 (2)	0.0143 (17)	−0.0015 (16)	0.0019 (14)	−0.0003 (14)
N5	0.0103 (17)	0.032 (2)	0.0126 (18)	−0.0012 (14)	0.0030 (13)	−0.0020 (15)
O1	0.0329 (19)	0.0201 (18)	0.0240 (16)	0.0030 (14)	0.0065 (14)	0.0001 (12)
O2	0.0302 (18)	0.0167 (16)	0.0308 (17)	−0.0035 (13)	−0.0004 (13)	0.0030 (13)
O3	0.0254 (18)	0.0261 (18)	0.0284 (18)	0.0080 (13)	0.0035 (14)	0.0016 (13)
O4	0.0290 (17)	0.0201 (18)	0.0238 (15)	−0.0087 (14)	0.0032 (12)	−0.0009 (12)
Co1	0.0135 (2)	0.0219 (2)	0.0160 (2)	−0.0002 (4)	0.00278 (15)	0.0001 (4)

### Geometric parameters (Å, °)

**Table:**

C1—N1	1.301 (6)	C12—C13	1.390 (5)
C1—C2	1.452 (8)	C12—H12	0.9500
C1—C3	1.503 (6)	C13—N5	1.324 (5)
C2—N2	1.294 (6)	C13—H13	0.9500
C2—C4	1.502 (6)	C14—C15	1.513 (5)
C3—H3A	0.9800	C14—Co1	2.008 (4)
C3—H3B	0.9800	C14—H14A	0.9900
C3—H3C	0.9800	C14—H14B	0.9900
C4—H4A	0.9800	C15—C16	1.538 (5)
C4—H4B	0.9800	C15—H15A	0.9900
C4—H4C	0.9800	C15—H15B	0.9900
C5—N3	1.304 (6)	C16—C17	1.514 (5)
C5—C6	1.480 (8)	C16—H16A	0.9900
C5—C8	1.482 (6)	C16—H16B	0.9900
C6—N4	1.292 (5)	C17—H17A	0.9800
C6—C7	1.480 (6)	C17—H17B	0.9800
C7—H7A	0.9800	C17—H17C	0.9800
C7—H7B	0.9800	N1—O4	1.342 (5)
C7—H7C	0.9800	N1—Co1	1.887 (4)
C8—H8A	0.9800	N2—O3	1.340 (5)
C8—H8B	0.9800	N2—Co1	1.884 (4)
C8—H8C	0.9800	N3—O2	1.361 (5)
C9—N5	1.349 (5)	N3—Co1	1.873 (4)
C9—C10	1.366 (5)	N4—O1	1.357 (5)
C9—H9	0.9500	N4—Co1	1.881 (4)
C10—C11	1.387 (5)	N5—Co1	2.061 (4)
C10—H10	0.9500	O1—H1	0.86 (2)
C11—C12	1.374 (5)	O3—H2	0.87 (2)
C11—H11	0.9500		
			
N1—C1—C2	112.7 (4)	C15—C14—H14B	107.0
N1—C1—C3	123.3 (5)	Co1—C14—H14B	107.0
C2—C1—C3	124.0 (4)	H14A—C14—H14B	106.8
N2—C2—C1	113.4 (4)	C14—C15—C16	111.2 (3)
N2—C2—C4	122.9 (5)	C14—C15—H15A	109.4
C1—C2—C4	123.7 (4)	C16—C15—H15A	109.4
C1—C3—H3A	109.5	C14—C15—H15B	109.4
C1—C3—H3B	109.5	C16—C15—H15B	109.4
H3A—C3—H3B	109.5	H15A—C15—H15B	108.0
C1—C3—H3C	109.5	C17—C16—C15	113.2 (4)
H3A—C3—H3C	109.5	C17—C16—H16A	108.9
H3B—C3—H3C	109.5	C15—C16—H16A	108.9
C2—C4—H4A	109.5	C17—C16—H16B	108.9
C2—C4—H4B	109.5	C15—C16—H16B	108.9
H4A—C4—H4B	109.5	H16A—C16—H16B	107.8
C2—C4—H4C	109.5	C16—C17—H17A	109.5
H4A—C4—H4C	109.5	C16—C17—H17B	109.5
H4B—C4—H4C	109.5	H17A—C17—H17B	109.5
N3—C5—C6	111.6 (4)	C16—C17—H17C	109.5
N3—C5—C8	124.5 (5)	H17A—C17—H17C	109.5
C6—C5—C8	123.9 (4)	H17B—C17—H17C	109.5
N4—C6—C5	112.2 (4)	C1—N1—O4	121.3 (4)
N4—C6—C7	124.3 (5)	C1—N1—Co1	116.1 (4)
C5—C6—C7	123.5 (4)	O4—N1—Co1	122.6 (3)
C6—C7—H7A	109.5	C2—N2—O3	121.6 (4)
C6—C7—H7B	109.5	C2—N2—Co1	116.1 (4)
H7A—C7—H7B	109.5	O3—N2—Co1	122.3 (3)
C6—C7—H7C	109.5	C5—N3—O2	119.8 (4)
H7A—C7—H7C	109.5	C5—N3—Co1	117.4 (4)
H7B—C7—H7C	109.5	O2—N3—Co1	122.8 (3)
C5—C8—H8A	109.5	C6—N4—O1	120.0 (4)
C5—C8—H8B	109.5	C6—N4—Co1	117.3 (3)
H8A—C8—H8B	109.5	O1—N4—Co1	122.7 (3)
C5—C8—H8C	109.5	C13—N5—C9	117.9 (4)
H8A—C8—H8C	109.5	C13—N5—Co1	121.4 (3)
H8B—C8—H8C	109.5	C9—N5—Co1	120.5 (3)
N5—C9—C10	122.8 (4)	N4—O1—H1	101 (4)
N5—C9—H9	118.6	N2—O3—H2	104 (4)
C10—C9—H9	118.6	N3—Co1—N4	81.38 (19)
C9—C10—C11	118.8 (3)	N3—Co1—N2	98.56 (11)
C9—C10—H10	120.6	N4—Co1—N2	178.50 (16)
C11—C10—H10	120.6	N3—Co1—N1	177.73 (16)
C12—C11—C10	119.1 (4)	N4—Co1—N1	98.26 (11)
C12—C11—H11	120.4	N2—Co1—N1	81.7 (2)
C10—C11—H11	120.4	N3—Co1—C14	91.91 (16)
C11—C12—C13	118.4 (4)	N4—Co1—C14	88.78 (17)
C11—C12—H12	120.8	N2—Co1—C14	89.72 (17)
C13—C12—H12	120.8	N1—Co1—C14	85.84 (16)
N5—C13—C12	123.0 (4)	N3—Co1—N5	90.94 (14)
N5—C13—H13	118.5	N4—Co1—N5	91.88 (14)
C12—C13—H13	118.5	N2—Co1—N5	89.62 (14)
C15—C14—Co1	121.2 (3)	N1—Co1—N5	91.32 (14)
C15—C14—H14A	107.0	C14—Co1—N5	177.14 (18)
Co1—C14—H14A	107.0		

### Hydrogen-bond geometry (Å, °)

**Table:**

*D*—H···*A*	*D*—H	H···*A*	*D*···*A*	*D*—H···*A*
O1—H1···O4	0.86 (2)	1.61 (2)	2.465 (3)	174 (6)
O3—H2···O2	0.87 (2)	1.60 (2)	2.465 (3)	171 (6)
